# 5-aza-2’-deoxycytidine induces apoptosis and inhibits tumour growth in vivo of FaDu cells, a specific HPVnegative HNSCC cell line

**DOI:** 10.1371/journal.pone.0253756

**Published:** 2021-09-17

**Authors:** Reem Miari, Naiel Azzam, Rinat Bar-Shalom, Fuad Fares

**Affiliations:** 1 Department of Human Biology, Faculty of Natural Science, University of Haifa, Haifa, Israel; 2 MIGAL Galilee Research Institute, Kiryat Shmona, Israel; Columbia University, UNITED STATES

## Abstract

Head and neck cancer squamous cell carcinoma (HNSCC) is the sixth most common cancer worldwide, resulting in over 600,000 new diagnoses annually. Traditionally, HNCC has been related to tobacco and alcohol exposure; however, over the past decade, a growing number of head and neck cancers are attributed to human papillomavirus (HPV) infection. 5-Aza-2’-deoxycytidine (5-AzaD) was demonstrated as an effective chemotherapeutic agent for acute myelogenous leukaemia. Preclinical data revealed that 5-aza inhibits growth and increases cell death of HPV(+) cancer cells. These effects are associated with reduced expression of HPV genes, stabilization of *TP53*, and activation of *TP53*-dependent apoptosis. The aim of the present study is to test the effect of 5-AzaD on growth of human squamous cell carcinoma (FaDu), a HPV(-) and p53 mutated cells, *in vitro* and *in vivo*. The effect of 5-AzaD on cell viability, cell cycle progression and induction of apoptosis was tested *in vitro*. The effect of 5-AzaD on tumour growth *in vivo* was tested using xenograft mice inoculated with FaDu cells. The results indicated that 5-AzaD reduced cell viability and induced apoptosis in FaDu cells *in vitro*. *In vivo* studies revealed that 5-AzaD suppresses the growth of tumours in xenograft mice inoculated with FaDu cells through inhibition of proliferation and induction of apoptosis. These findings may emphasis that 5-AzaD is effective in treatment of HPV(-) HNSCC tumours through *TP53* independent pathway. Future studies are needed in order to clarify the molecular mechanism of action of 5-AzaD in HPV(-) cancer cells.

## 1. Introduction

Head and neck cancer comprise a group of tumours that are anatomically located in the oral cavity, the oropharynx, the nasal cavity, paranasal sinuses, the nasopharynx, the hypopharynx and the larynx. Most of these cancers (90%) exhibit a histology of squamous cell carcinoma and therefore, are called head and neck squamous cell carcinoma (HNSCC) [1, 2]. HNSCC constitutes a major public health burden with an annual incidence of almost 600,000 patients worldwide and a mean of 5-year survival rate of less than 50% [3]. In the United States, 50,000 cases are diagnosed each year and nearly 10,000 deaths are attributable to this disease group [4]. Although the risk factors include tobacco and human papilloma virus (HPV), there is only an elemental understanding of the molecular, cellular and environmental mechanisms that drive HNSCC pathogenesis, and there are only limited therapeutic options [5].

The current standard treatment for advanced HNSCC is concurrent platinum-based chemo-radiotherapy. Despite encouraging results, treatment is still associated with significant toxicity and frequent loco-regional recurrences [6]. The development of malignant tumour of HNSCC is the consequence of multi-step process driven by accumulation of genetic alterations. These alterations include inactivation or loss of function of tumour suppressor genes as well as activation or gain of function of proto-oncogenes. In addition, reactivation of Telomerase, which is associated with telomere maintenance and immortalization might also be involved [7]. Accumulated scientific evidences suggest that epigenetic alterations, including DNA methylation, histone covalent modifications, chromatin remodelling and non-coding RNAs, are frequently involved in oral carcinogenesis, tumour progression, and resistance to therapy. Furthermore, epigenetic modifications also contribute to cellular plasticity during tumour progression and to the formation of cancer stem cells (CSCs), [8].

U.S. Food and Drug Administration (FDA) and the European Commission (EC) has approved DNA demethylations agents 5-azacytidine (5-aza) and its structural analogue, 5-aza-2’-deoxycytidine (5-AzaD), for use in the therapy of myelodysplastic syndromes (MDS) and acute myelogenous leukaemia (AML) [9, 10]. Moreover, it has been reported that 5-AZaD inhibits metastatic spread and cell proliferation in the 1833 xenograft model of breast cancer [11]. On the other hand, treatment with 5-AZaD resulted in global demethylation and significantly increased XPC mRNA expression in human melanoma cell lines [12]. It was reported that 5-AZaD has analgesic efficacy in a metastatic bone cancer through activation of an endogenous analgesic mechanism and it may show promise for management of pain related to the malignancy [13].

5-aza inactivates DNA methyltransferase-1 (DNMT-1), resulting in global demethylation of the cellular genome [13]. The link between demethylation-induced transcription and the therapeutic effects of these drugs is not well-established [14]. Demethylation can re-activate tumour suppressors that were repressed by promoter methylation, resulting in tumour suppressor-induced cancer cell death [15]. Moreover, 5-aza and 5-AzaD cause DNA damage and activate the DNA damage response in some cells. However, the nature of this DNA damage has not been fully characterized and is likely to be cell type-dependent [16].

Clinical trial and experimental data using xenografed tumors indicated that 5-aza induced growth inhibition and cell death in HPV(+) HNSCC associated with reduced expression of HPV genes and activation of p53-dependent apoptosis [17, 18]. Similarly, treatment of HPV-transformed cervical and head and neck cancer cells with 5-azaD reduced expression of HPV genes (E6 and E7) and increased target proteins including *TP53* and p21 resulted in reduction of proliferation rates [19]. Although *TP53* is mutated in 84% of HPV(-) HNSCC tumours, it is rarely mutated in HPV^+^ HNSCC and is alternatively suppressed by the HPV E6 viral oncoprotein [20]. In our study, we assessed the effect of 5-AzaD on growth and viability of FaDu cells, a head and neck cancer cell line originated from hypopharyngeal carcinoma. These cells are known to be HPV negative and *TP53* mutated [21].

## 2. Materials and methods

### 2.1 Cell culture

The human epithelial FaDu cancer cell line and normal human dermal fibroblasts (NDHF) were obtained from the American Type Cell Collection (Manassas, VA, USA). Histological examination classified FaDu cells as squamous cell carcinoma of laryngopharynx Grade II. FaDu cells are poorly differentiated, HPV(-) and p53 mutated. Cells were grown in MEM (Minimum Essential Medium (Thermo Fisher, USA) and supplemented with 10% Fetal bovine serum, 50 IU/ml ampicillin, 50 μg/ml streptomycin and 2 mM L-glutamine. All cells were maintained at 37°C in 5% CO_2_.

### 2.2 Cell proliferation assay-XTT

To assess the effect of 5-AzaD on cell viability, FaDu and NDHF cells were seeded in eight plicate, in 96-well plates at a density of 5 x 10^3^ cells/well in 100 μl of culture medium. Cells were treated with 0.5–10 μM of 5-AzaD for 48 hours (h), or with 5 μM for 24, 48 and 72 (h). Cell viability was determined by XTT Cell Proliferation Kit (Biological Industries, Israel), according to the manufacturer’s instructions. Results were calculated as percentages of the proliferation of untreated control cells that was defined as 100%.

### 2.3 Cell cycle analysis

FaDu cells were treated with 5 μM of 5-AzaD for 24, 48 and 72 h. Untreated FaDu cells were exposed to 0.01% DMSO. At the end of treatment, cells were, washed with PBS and fixed with 70% ethanol for one hour (h). Following fixation, cells were treated with 0.1% NP-40 for 5 minutes in 4°C and incubated on ice with 100 μg/mL RNase for 30 minutes. Finally, 50 μg/mL of propidium iodide (PI) was added for 20 minutes on ice. DNA content was examined by flow cytometer (BD FACS Canto II) (Becton Dickinson, USA).

### 2.4 Annexin-V/PI double-staining assay

Apoptotic cell death was evaluated and quantified by flow cytometry based on Annexin-V FITC and PI double staining kit (Mebcyto^®^ Apoptosis Kit, MBL, Japan), according to the manufacture procedure. Briefly, FaDu cells were treated with 5 μM of 5-AzaD for 24, 48 or 72 h. Untreated FaDu cells were exposed to 0.01% dimethyl sulfoxide (DMSO). Cells were washed and suspended in ice-cold PBS and then resuspended in ice-cold binding buffer containing FITC-conjugated Annexin-V and PI. Subsequently, cells were analysed by flow cytometer (BD FACS Canto II) (Becton Dickinson, USA).

### 2.5 Western blot analysis

FaDu cells were treated with 5 μM of 5-AzaD for 24–48 h, while control cells were treated with 0.01% DMSO. Protein extraction was performed using RIPA buffer according to manufacturer instructions. 60 μg of total proteins were separated on SDS-PAGE and then transferred to a 0.45-micrometer-pore-size nitrocellulose membrane. Membranes were blocked with 5% milk TBST solution and incubated with primary antibodies: monoclonal rabbit anti-caspase 3, 8 and 9 (Abcam, Cambridge, UK); polyclonal rabbit anti-cleaved PARP (Asp214) (Cell Signalling, Danvers, MA, USA); monoclonal rabbit anti-Cytochrome C antibody (Abcam, USA) and monoclonal mouse anti-β-actin (Santa Cruz Biotechnology, CA, USA) that serves as internal control. For reference, the membranes were washed three times with TBST and incubated with anti-rabbit secondary antibody conjugated to horseradish peroxidase or anti-mouse HRP secondary antibody (Jackson Immune Research Laboratories and DAKO, Glostrup, Denmark) for 1 h at room temperature. Following three washes, the antigen/antibody complex was detected with the (ECL kit WesternBright^TM^ ECL detection reagent (Advansta, San Jose, CA, USA) using the Amersham Imager 600 (GE, USA).

### 2.6 Xenograft model for head and neck cancer

The *in vivo* study was approved by the institutional animal experimental ethical committee at University of Haifa (Ethics Number: 596/18). Seven-week old athymic nude male mice (ENVIGO, Israel) were maintained in temperature- and humidity-controlled rooms with a 12 h light/dark cycle, and given food and water ad libitum throughout the course of the experiment. Xenograft animal model was generated via subcutaneous injection of 1x10^6^ FaDu cells in 1:3 Cultrex® Basement Membrane Extract (BME) (Trivagen, Gaithersburg, MD) in the right dorsal flank of nude mice. Eight days post implantation, when the tumours reached a volume of ~200 mm^3^, mice were divided into 2 groups (8 mice in each group). Mice were intraperitoneal (IP) injected three times a week for 3 weeks with either PBS X 1 for the control group, or with 2.5 mg/kg of 5-AzaD for the experimental group. During the experiment, tumour volumes and body weight were measured twice a week. Tumor size was measured biweekly with a caliber, and the volumes were calculated using the formula: length x width^2^ x 0.52. At the end of the experiment, mice were sacrificed, the tumour tissues were dissected and the final volumes and weights were measured and tested for histological studies.

### 2.7 Immunohistochemistry (IHC) and histological analysis

For IHC analysis, tumours were excised from mice as described above and fixed with paraformaldehyde. 4 μm paraffin sections were prepared for histology analysis (4 sections for each tumour tissue). Standard protocols of deparaffinization and antigen retrieval were used. The tissue slides were stained with haematoxylin and eosin (H&E) and with mouse monoclonal antibodies to Ki67 and active caspase 3 antigen using standard protocols (Patho-Lab Diagnostics Ltd, Rehovot, Israel).

### 2.8 Statistical analysis

All data were expressed as mean value ± Standard Error (SE). Statistical analyses were performed, using Student’s–*t—*test for comparison between two groups. The SPSS software served for calculation of differences. Probability values of *P<0.05, **P<0.01 and ***P<0.001 were considered statistically significant.

## 3. Results

### 3.1 5-AzaD inhibits human head and neck cancer cell growth in a time and concentration dependent manner

**Fig 1:**
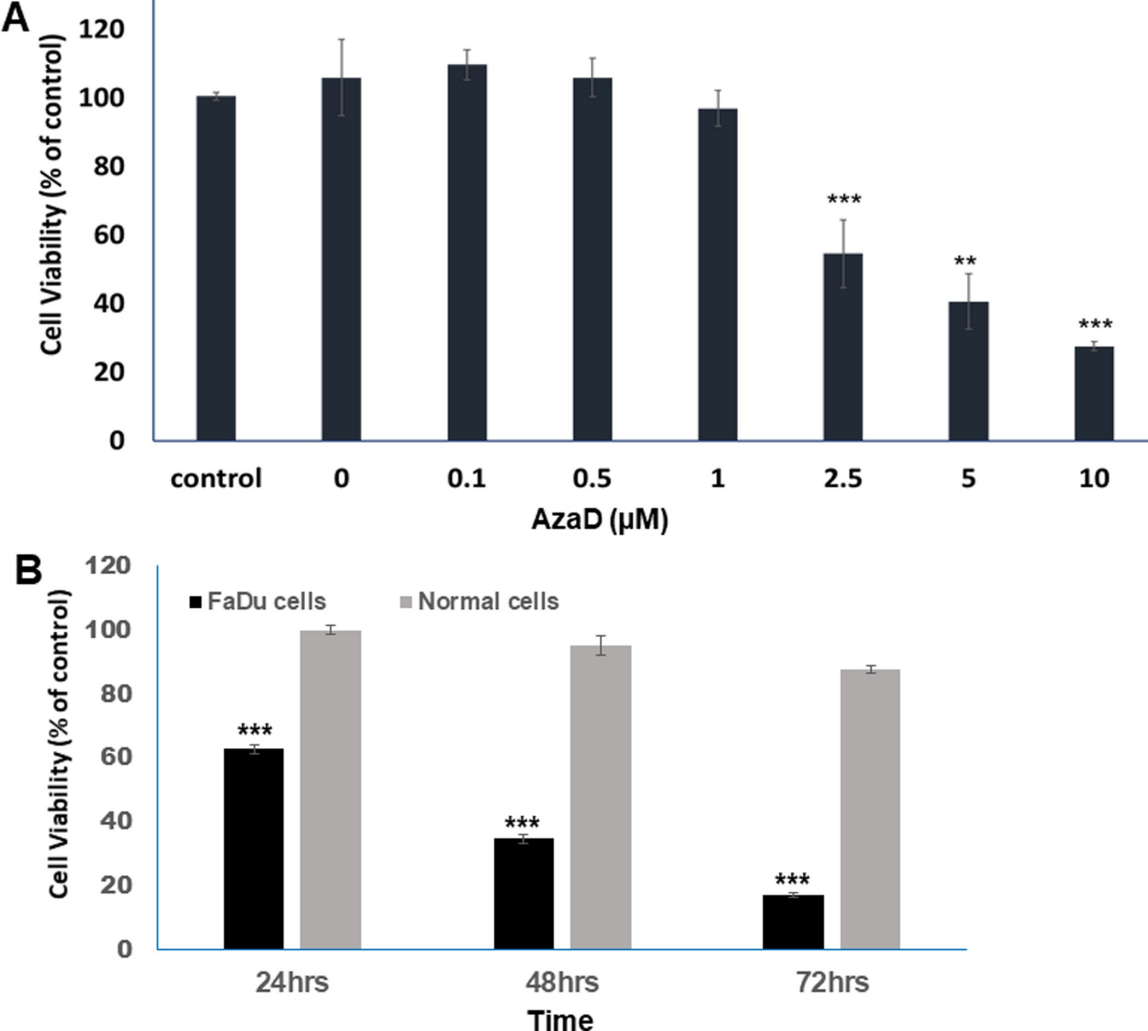
Cell viability following 5-AzaD treatment. Cell viability was detected by XTT following treatment of FaDu cells with increasing concentrations of 5-AzaD (0, 0.1, 0.5,1,2.5,5 and 10 μM) for 48 h (A). FaDu and normal fibroblast cells (NDHF) were treated with 5 μM of AzaD for a period of 24, 48 and 72 h (B). Untreated control cells, FaDu and NDHF, were exposed to 0.01% DMSO. Data were presented as mean ± SD of three independent experiments in which each treatment was performed in eight replicates, and are expressed as percentages of the respective untreated control cells that was defined as 100%. Statistical significance was determined by two-tailed Student’s *t*-test. Significance is indicated as **P<0.01, ***P<0.001.

Treatment with 5-AzaD was found to decrease the viability of FaDu cells in a concentration and time dependent manner. The effect of 5-AzaD on cell viability was measured using the XTT method. FaDu cells were treated with increasing concentrations of 5-AzaD (0.1–10 μM) for 48. Overall, treatment with 0, 0.1, 0.5 and 1 μM of 5-AzaD for 48 h did not elicit significant changes in cell viability. However, a concentration dependent effect was demonstrated when cells were treated with 2.5, 5 and 10 μM for 48 h resulted in a significant (P<0.001) reduction of cell viability to 54%, 40% and 27%, respectively (Fig 1A). Moreover, cell viability was tested following treatment with 5 μM of 5-AzaD for 24, 48 and 72 h and results indicated that viability of FaDu cells was reduced in a time dependent manner, to 62.7%, 34.67% and 17.2%, respectively. Interestingly, treatment with 5 μM of 5-AzaD has no significant effect on cell viability of normal NDHF cells (Fig 1B).

### 3.2 5-AzaD induces sub-G1-phase accumulation of FaDu cells

**Fig 2:**
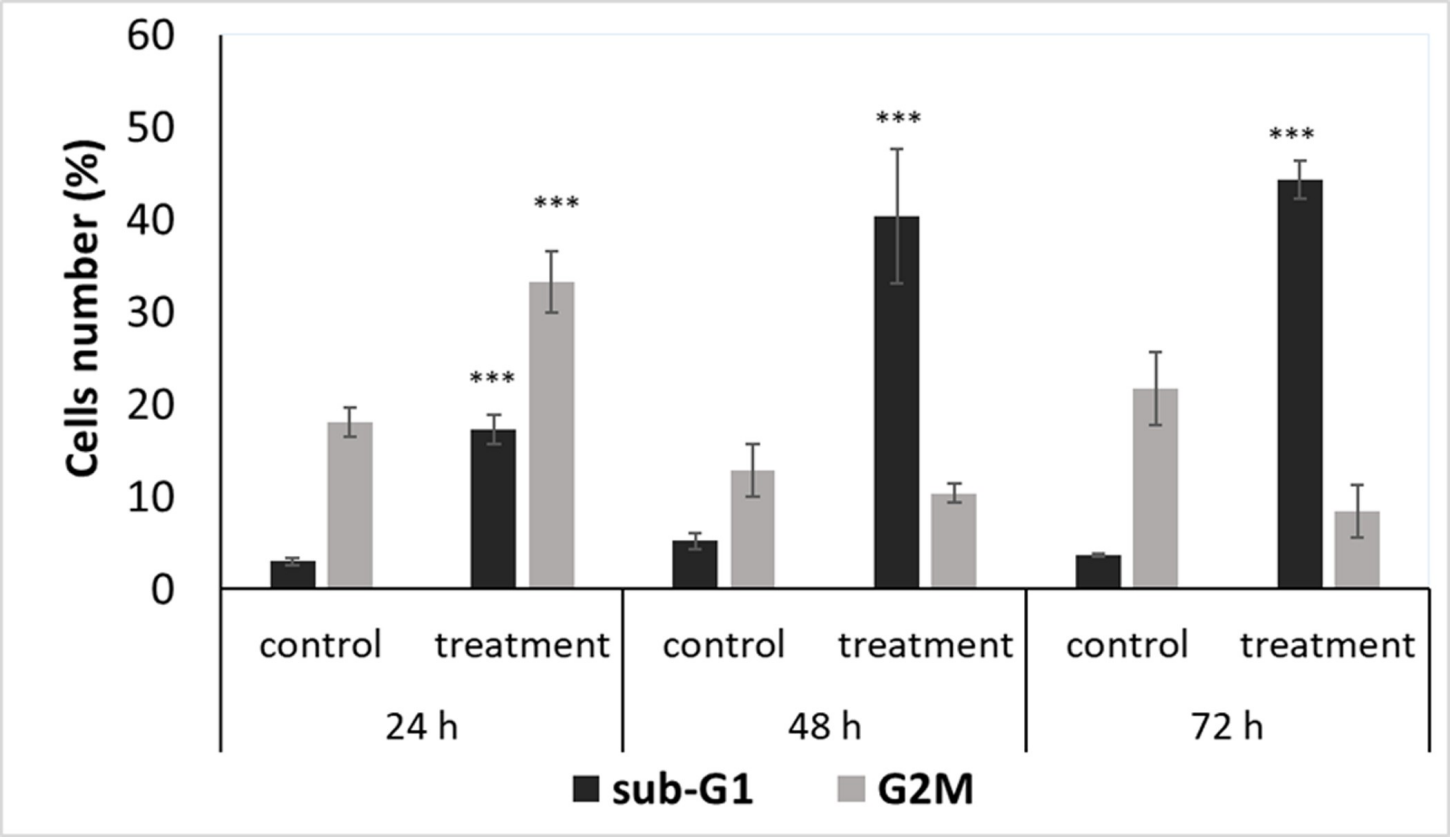
Cell cycle analysis of FaDu cells following 5-AzaD treatment. FaDu cells were treated with 5 μM of 5-AzaD for 24, 48 and 72 h; Untreated FaDu cells (control) were exposed to 0.01% DMSO. Cells were subjected to cell cycle analysis by flow cytometry as described under “Materials and methods”. The graph summarize percentages of cells in sub-G1 and G2/M phases following treatment. The results are presented as mean ± SD of four experiments each conducted in duplicates and are expressed as percentages from total of 10,000 analysed cells. Statistical significance was determined by two-tailed student’s *t*-test (treatment VS. control) and is indicated as *p < 0.05; **p < 0.01; ***p < 0.001.

In order to study the effect of 5-AzaD on cell cycle progression, FaDu cells were treated with 5 μM of 5-AzaD for 24, 48 and 72 h. The distribution of treated cells in the different phases of cell cycle was determined by FACS analysis as described under ‘‘Materials and Methods”. The results indicated that exposure of the cells to 5 μM of 5-AzaD for 24 h resulted in a significant accumulation of the cells in G2/M phase as compared to untreated cells (18.3% ± 1.6% in control vs. 33.3% ± 3.4% in treated cells). Moreover, extended periods of treatment to 48 and 72 h resulted in accumulation of the cells in sub-G1 phase, suggesting the occurrence of cell death. Sub-G1 levels at 24, 48 and 72 h post-treatment were 17.3% ± 1.5, 40.3% ± 7.2% and 44.3% ± 2%, respectively, whereas sub-G1 amount of untreated cells was approximately 3% ± 0.4% (Fig 2).

### 3.3 5-AzaD induces apoptosis in FaDu cells

**Fig 3:**
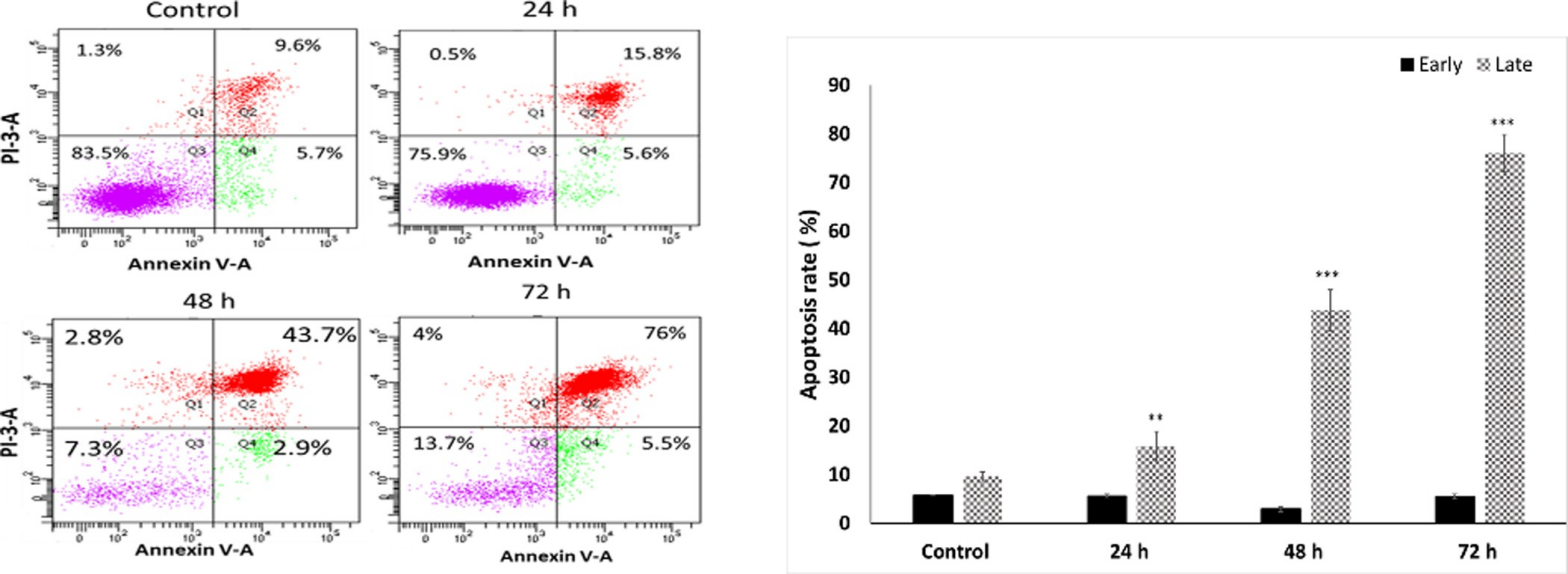
Measurement of apoptotic cells using an Annexin V binding. FaDu cells were treated with 5 μM of 5-AzaD for 24, 48, 72 h. Untreated FaDu cells were exposed to 0.01% DMSO. Analysis of Annexin V-FITC/PI double-stained was performed using flow cytometry. In a representative plot (A), the power left quadrant (Q3) represents viable cells, the upper left quadrant (Q1) indicates necrotic cells, the lower right quadrant (Q4) denotes early apoptotic cells and the upper right quadrant (Q2) represents late apoptotic cells. The percentage of Annexin V-FITC positive cells from the early and late stage of apoptosis following treatment for 24, 48 and 72h is shown. Data are presented as mean ± SE of three independent experiments, each conducted in duplicates [mean (B)]. Statistical significance was determined by two-tailed student’s *t*-test [treatment vs. control (untreated cells)] and is indicated as **p < 0.01, ***p < 0.001.

Annexin V-FITC binding analysis and Propodeum Iodide (PI) staining were performed to quantify cell death arising from apoptosis or necrosis. The apoptotic cells were counted as early apoptotic cells (quadrants Q4) and late apoptotic cells (quadrants Q2), and represented as percentage of apoptotic cells from total cell population. Representative experiment is shown in Fig 3A. The number of intact cells was significantly decreased following treatment with 5-AzaD comparing to the control cells as shown in Fig 3A. Treatment with 5-AzaD significantly increased (P<0.01) the number of Annexin V-FITC positive/PI-positive cells. The percentages of cells (Q2+Q4) from three independent experiments after treatment with 5 μM of 5-AzaD for 24, 48 and 72 h were: 45.85% ± 8.838, 49.75% ± 7.99 and 78.5% ± 3.9, respectively, vs. 7.2% ± 0.989 of untreated FaDu cells. Fig 3B indicates the percentage of Annexin V-FITC positive cells from the early (Q2) and late (Q4) stages of apoptosis following treatment for 24, 48 and 72h.

### 3.4 5-AzaD induces apoptosis in FaDu cells by activation of caspases

**Fig 4:**
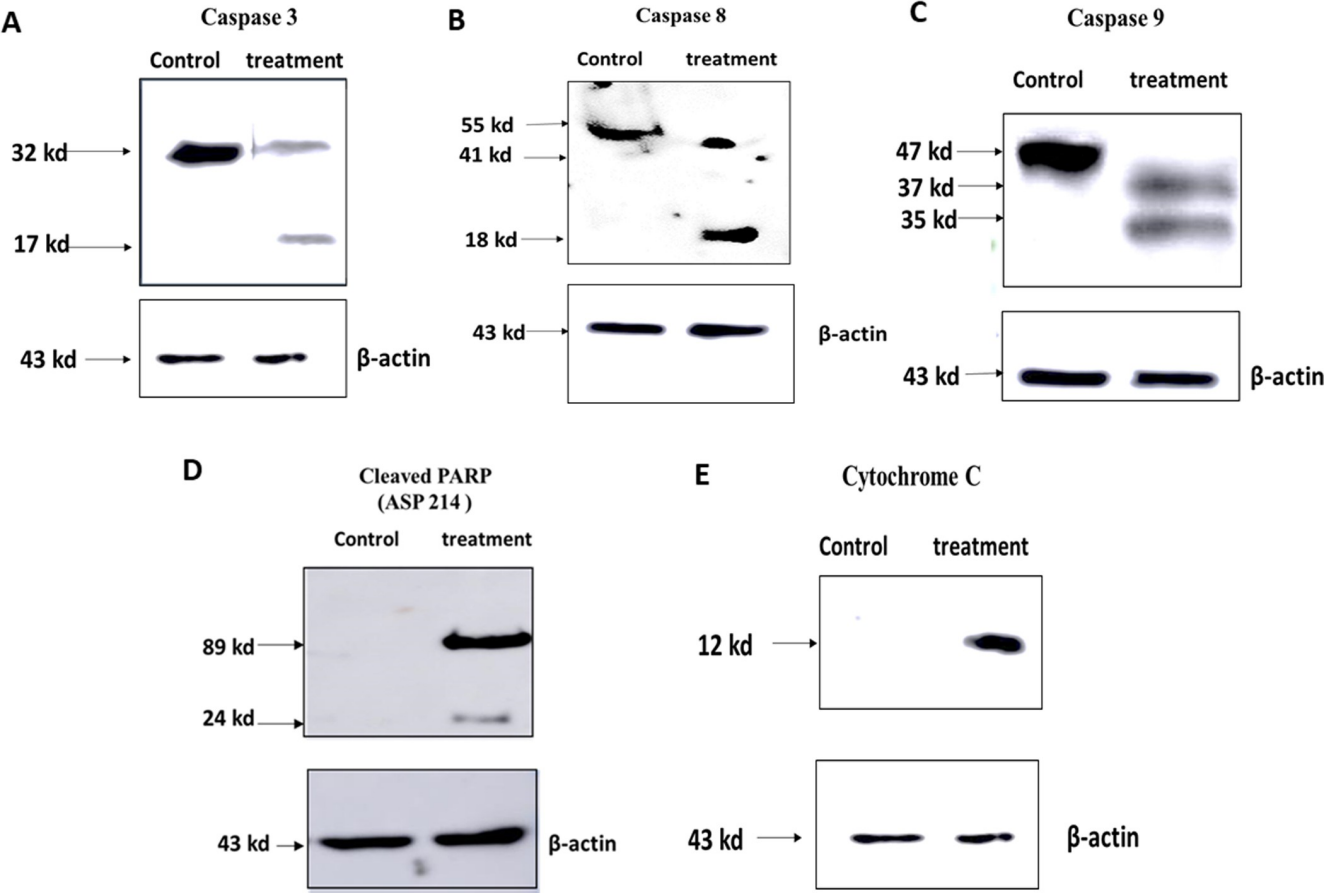
Activation of caspases following 5-AzaD treatment. FaDu cells were treated with 5 μM of 5-AzaD for 48 h. Untreated FaDu cells were exposed to 0.01% DMSO. Total protein was extracted and 60-μg was separated on SDS-PAGE and transferred to nitrocellulose membranes. Caspase 3 (A), Caspase 8 (B), Caspase 9 (C), Cleaved PARP (D), and Cytochrome C (E) were detected by using specific primary antibodies. For equalization of protein amounts, mouse anti-human β-actin monoclonal antibody was used. The figure shown is representative of three independent experiments. Results were analysed with Amersham Imager 600.

To further investigate the mechanism of action of cell death induced by 5-AzaD treatment, a western blot analysis was performed to detect proteins that have been shown to be involved in both extrinsic (caspase 8) and intrinsic (Cytochrome-c and caspase 9) apoptosis pathways (Fig 4).

Cells were treated with an effective concentration of 5-AzaD (5μM) for 48 h. Subsequently, cells were lysed and subjected to western blot analysis using antibodies for caspase 3 (Fig 4A), caspase 8 (Fig 4B), caspase 9 (Fig 4C), cleaved PARP (Fig 4D) and cytochrome-C (Fig 4E). Results indicated the appearance of the cleaved active subunit of caspase 3 (17KD), caspase 8 (43 kDa), caspase 9 (35 kDa), active cleaved PARP as well as release of Cytochrome-C. The equal actin reference levels in the untreated and treated samples indicated actual differences (Fig 4).

### 3.5 5-AzaD inhibit tumour growth in xenograft mouse model

**Fig 5:**
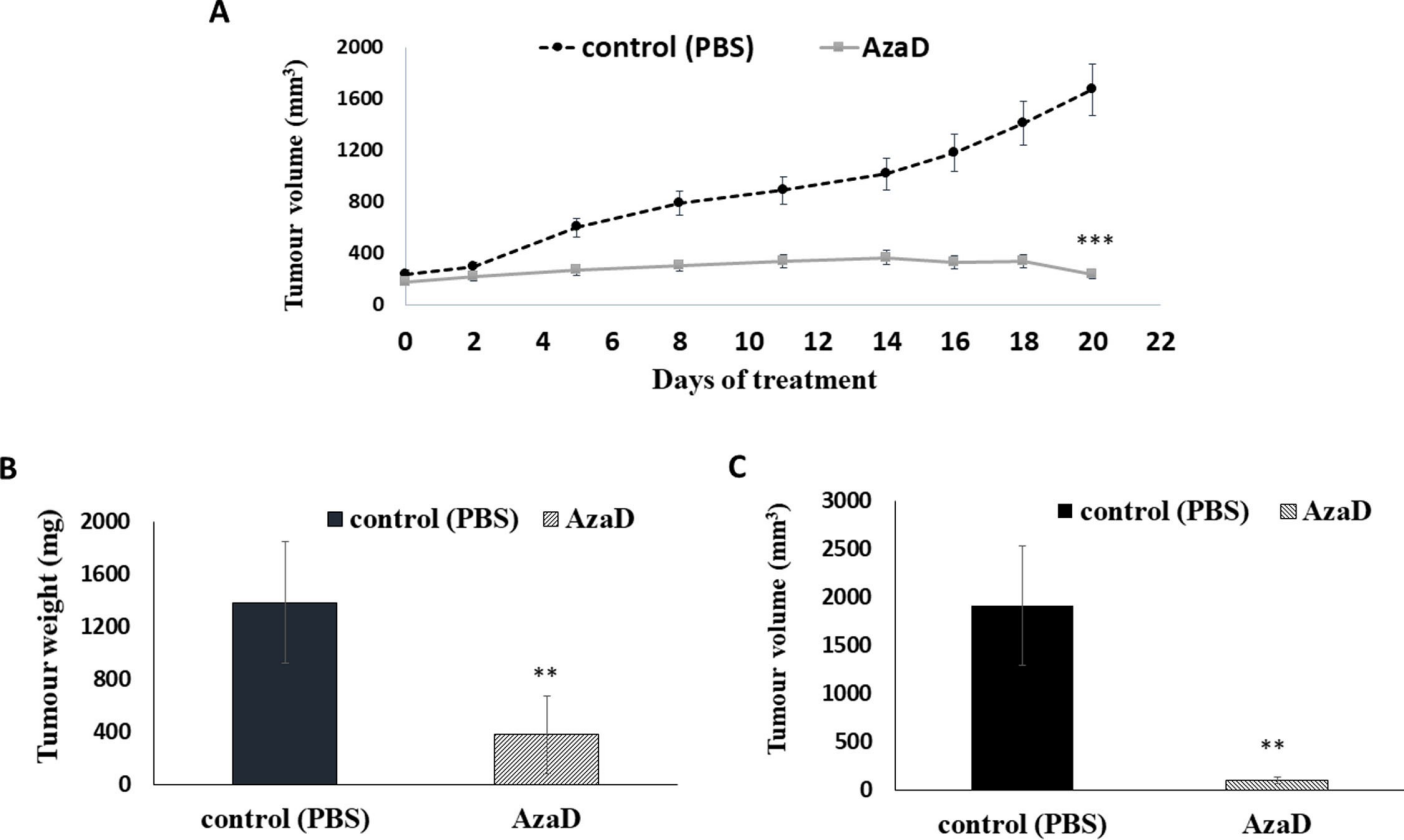
The effect of 5-AzaD on tumour development using xenograft mice. Athymic nude male mice were SC implanted with 1X10^6^ FaDu cells. When the tumours reached a volume of ~200 mm^3^ the mice were divided into 2 groups (n = 8). Mice were intraperitoneal (IP) injected three times a week either with PBS X 1 to the control group, or with 2.5 mg/kg of 5-AzaD for 3 weeks. During the experiment, tumour volumes were measured twice a week using calibre meter (A). On day 21 at the end of the experiments, mice were sacrificed, and the tumour tissues were dissected and measured for weight (B) and volume (C). The results were presented as the mean± SD (n = 8). Statistical significance was determined by two tailed student’s–*t–*test and assigned as **P < 0.01, ***P < 0.001. In order to better understand how 5-AzaD treatment affects tumour growth *in vivo*, tumours were fixed and sectioned for histological staining. H&E staining indicated that 5-AzaD treatment induced apoptosis of tumour cells and inhibited mitosis when comparing to the control group (Fig 6A). Morphological criteria for diagnosis of mitosis, using the H&E staining, included reporting of increased mitosis above or below the normal background levels, of what is usually seen in control animals [22].

To investigate the activity of 5-AzaD against HNSCC tumours *in vivo*, mice bearing FaDu xenografts were treated with 2.5 mg/kg three times a week for 3 weeks. Treatment with 5-AzaD resulted in a significant suppression of tumour growth in mice as shown by the measurement of tumour volumes (Fig 5A). Mice were sacrificed three weeks post treatment and tumours were collected, measured and weighed. The average tumour weight from control animals was ~5 times higher than that from treated animals (Fig 5B). Similarly, the tumour volume in the control group increased by ~9 folds compared to the volume of the tumours from the treated animals (Fig 5C).

The morphological criteria for diagnosis of apoptosis using the H&E stained slides, includes single cells or small clusters of cells; cell shrinkage nuclear shrinkage, karyorrhexis; hyper eosinophilic cytoplasm; nuclear pyknosis; apoptotic bodies; body macrophages with engulfed cytoplasmic apoptotic; no inflammation; decreased cellularity in the tissue whether the process is moderate or severe [23, 24].

**Fig 6:**
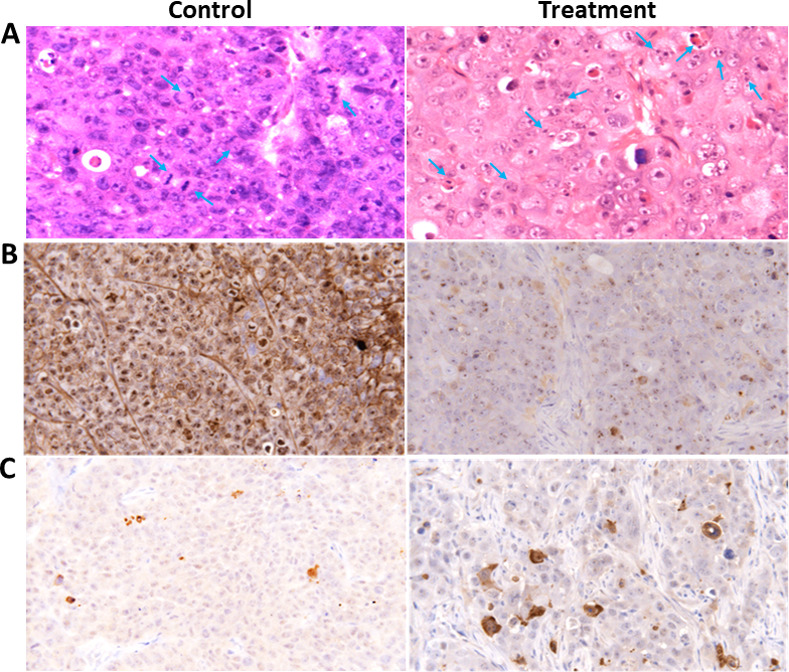
H&E stained images and Ki-67 and active caspase 3 immunostaining of tumour sections from control and treated animals. Nude mice bearing FaDu tumour cells were treated with 2.5 mg/kg of AzaD or PBS 3 times a week for 3 weeks. At the end of the experiment, mice were sacrificed and the tumour tissues were dissected. (A) Representative H&E stained images of isolated tumours are shown. Mitotic cells in the control and apoptotic cells in the treated groups are marked with arrows. (B) Proliferation profiles of the cells were detected by Ki-67 immunostaining as described under “Material and Methods”. The images are representatives of the results obtained from control and treatment groups (Magnification, 400X). (C) Apoptotic cells were detected by caspase 3 immunostaining positivity; criteria are explained in the materials and methods section. The images are representatives of the results obtained from control and treatment groups.

Tumour sections were stained with Ki-67 as a marker of proliferation. 5-AzaD was found to inhibit significantly (**P<0.01) proliferation of FaDu xenografts (Fig 6B). In addition, 5-AzaD was revealed to induce apoptosis by activation of caspase-3 in FaDu xenografts as measured by the number of cells expressing active caspase-3 in tumor sections from treated compared to untreated animals (Fig 6C).

Qquantitative analysis of Ki67 and caspase 3 on tumor sections was shown in Table 1.

**Table 1:** Qquantitative analysis of Ki67 and caspase 3 on tumor sections.

**Score**	**Descriptor**	**Description**
0	None	No labeling
1	Minimal	Very pale
2	Mild	Pale brown
3	Moderate	Brown
4	Marked	Dark brown
5	Intense	Very dark brown
	**Control (n = 16)**	**Treatment (n = 16)**
Ki67	4.7±0.42	1.8±0.5 **
Caspase 3	1.01±0.2	4.2±0.50 **

**P<0.01.

**Fig 7:**
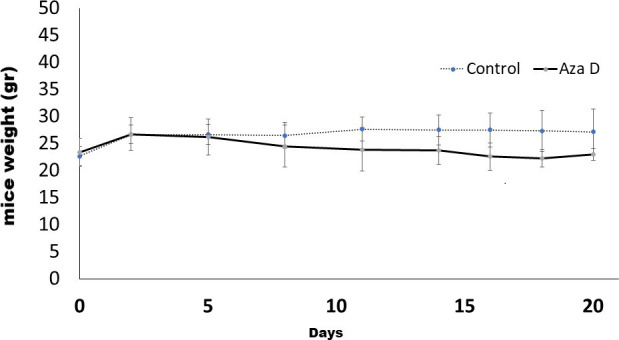
The effect of 5-AzaD on body weight. Body weight of control and treated animals was measure twice a week through the experiment. Data are presented as Mean ± SE.

Treatment of the animals with 5-AzaD for three weeks resulted in non-significant decrease in body weight (Fig 7).

## 4. Discussion

The DNMT inhibitors, 5-azacytidine (5-aza) and its structural analogue, 5-aza-2’-deoxycytidine (5-AzaD), have been approved for treatment of myelodysplastic syndrome. These inhibitors are being tested in clinical trials for Acute myeloid leukaemia (AML), Chronic myeloid leukaemia (CML) and are designated as a promising therapeutic possibility for haematological malignancies [25, 26].

In regards to HPV and cancer, Biktasova and colleagues [27] indicated that 5-aza decreased the expression of all HPV16 genes, including the major oncoproteins E6 and E7 in HPV(+) HNSCC. This was accompanied by increase of *TP53* expression and induction of apoptosis as detected in pre-clinical animal models following 5–7 days of 5-aza treatment. These data suggest that demethylation therapy might be effective for treating HPV(+) HNSCC through activation of *TP53* pathway, although, HPV negative HNSCC was not examined in this regard. *TP53* mutations that accompany with loss-of-function are the most frequent gene mutations detected in HNSCC HPV (-). On the other hand, *TP53* mutation is rarely seen in HPV(+) HNSCC, likely because of the role of E6 protein that plays in promoting proteasomal degradation of wild-type p53 [28]. The effect of 5-AzaD on cell viability occurred in time and concentration dependent manners with IC_50_ of 2.5 μM. The present findings match those of previous studies regarding the effects of 5-aza on numerous cancer types, e.g., breast, oesophageal, colorectal and anaplastic large cell lymphoma [29, 30]. Interestingly and as detected in our experiments, 5-AzaD had no significant effect on viability of normal human fibroblast cells (NDHF). These results are in agreement with recent studies regarding normal cells, where 5-aza has been shown to affect only rapidly dividing cancerous cells and not non-proliferating normal cells [31, 32].

Treatment of FaDu cells with 5-AzaD for 24 h resulted in a cell cycle arrest at G2/M phase. This result is in agreement with Alexander et al. [33] report, which showed that 5-aza induced cell cycle arrest in neuroendocrine carcinoid types.

The cell cycle in eukaryotes is regulated by cyclin dependent kinases (CDKs). Sequential formation, activation, and subsequent inactivation of cyclins and CDKs are critical for the control of cell cycle [34, 35]. Cyclin-dependent kinase inhibitors (CDKIs) play a key role in controlling cell cycle progression by negatively regulating the CDK activities at an appropriate time during the cell cycle [36, 37]. Moreover, *TP53* is a tumour suppressor gene that functions especially as a transcription factor by either activating or down regulating gene expression, leading to cell cycle arrest or apoptosis [38]. Recently, scientists showed that cell cycle arrest could also occur through *TP53* independent pathway. Wahl et al., [39] showed that cell cycle arrest at G2/M phase correlated with low levels of *TP53*. It was suggested that the anticancer agent paclitaxel (Taxol) induces a G2/M arrest followed by p53-independent apoptosis [39]. Moreover, Seol and colleagues [40] found that As_2_O_3_ inhibits the proliferation of head and neck cancer cells, PCI-1 and PCI-13 (*TP53* mutated), via G2/M arrest through induction of p21 and reduction of cdc2 kinase activity [39, 40]. These findings are in agreement with the results found in the present study, where treatment of FaDu cells (*TP53* mutated) with 5-AzaD caused G2/M arrest followed by induction of apoptosis.

Extension of the treatment periods (48–72 h) with 5-AzaD increased the accumulation of cells in Sub-G1, a Phase that is often related to non-living cells [41]. The Annexin V/PI assay, detection of cytochrome-C, caspases and PARP confirmed the occurrence of apoptotic cell death following treatment with 5-AzaD. The biochemical and molecular pathways involved in apoptosis can be generally grouped as “private” pathways and “common” pathways. The “private” pathways are executed through diverse signal cascades, which converge at the mitochondrial level [42]. Comparably, the “common” pathways converge together on a set of molecular components collectively known as caspases. These components activate a cascade of proteolytic events leading to DNA fragmentation. Among these components are various chemical agents and UV radiation that induce activation of pro-caspase-8 via FasL–FADD or activation of Apaf-1/caspase 9 via induction of p53 → Bax → cytochrome *c*. This is followed by activation of caspase 3 and inactivation of PARP, ultimately leading to cell death (apoptosis) [43]. Previously we found that treatment of SCC-9 and SCC-25, HNSCC cell lines, with zebularine, a methyltransferase inhibitor, induces cell cycle arrest in G2/M and Sub-G1, respectively, followed by induction of apoptosis [44].

In the present study, we have demonstrated that 5-AzaD has a potential beneficial effect on inhibition of tumour growth of subcutaneously transplanted FaDu cells in nude mice. Treatment of xenografts with 5-AzaD for three weeks resulted in a significant inhibition of tumour growth compared to untreated animals. Immunohistochemical and histological studies indicated that 5-AzaD inhibits cell proliferation as detected by Ki67 staining. Possibly these effects may occur through inhibitory effects of 5-AzaD on blood vessel formation. It is well known that genomic methylation and dimethyl transferase activity are increased in tumour endothelial cells leading to angiogenesis and cell growth. This may mediated by inactivation of angiogenesis inhibitory genes such as; thrombospondin-1 (TSP1) and insulin-like growth factor binding protein-3 (IGFBP3) genes [43, 45, 46]. These observations are in agreement with previous studies, which reported that Methyltransferase inhibitors (5-AazD or zebularine) decreased vessel formation and inhibited angiogenesis in different tumour models [47]. In addition, it is well known that DNA methylation is the most important epigenetic phenomena that leads to inactivation of several key tumour suppressor genes required to drive the initiation and progression of cancer [48]. Moreover it has been shown that inhibition of DNMT1 induced apoptosis, accompanied by the loss of mitochondrial membrane potential, Bcl-2 reduction, and activation of Bax, *TP53*, caspase-3, and caspase-8, in lung cancer cells [49]. Other studies reported that inhibitors of DNMT1 induce *TP53*-independent endoplasmic reticulum stress and autophagy in colorectal cancer cells [50]. Other study indicated that 5-AzaD inhibited tumour growth and metastasis of osteosarcoma [51]. These observations may indicate that the molecular mechanism of DNMT inhibitors and the pathways lead to growth inhibition and apoptosis are tissue specific. Moreover, it seems that 5-AzaD molecular mechanism of action in HPV(+) is different from that in HPV(-) cells.

It was reported that HNSCCs are an aggressive, genetically complex and difficult to treat group of cancers. The knowledge of genomic, proteomic, microbiomic and metabolomic alterations in HNSCCs is helping to move to personalised therapy, where each subtype can be treated as a separate disease [52].

Thus, understanding the molecular mechanism of 5-AzaD in head and neck cancer is necessary to enhance its efficacy and enable further development in treatment of cancer.

In conclusion, our findings indicated that 5-AzaD inhibits the growth and proliferation of head and neck cancer cells and induces apoptosis *in vitro* and *in vivo*. This may lead to the development of new strategies for the treatment of human head and neck cancer in advanced stages, for which at present there is no effective life-prolonging therapy.

## Supporting information

S1 Raw images (PDF)

S1 Data (XLSX)

S2 Data (XLSX)

S3 Data (XLSX)

S4 Data (XLSX)

S5 Data (XLSX)

## List of abbreviations

5-AzaD: 5-Aza-2’-deoxycytidine
5-aza: 5-azacytidine
°C: degrees centigrade
DMSO: dimethyl sulfoxide
DNA: deoxyribonucleic acid
DNMT-1: DNA methyltransferase–1
FDA: Food and Drug Administration
h: hours
H&E: haematoxylin and eosin
HNSCC: head and neck cancer squamous cell carcinoma
HPV: human papillomavirus
IHC: Immunohistochemistry
IP: intraperitoneal
M: molar
μg: microgram
μl: microliter
PI: propidium iodide
SD: Standard Deviation
SE: Standard Error
