# Supplementary material for: 5-aza-2’-deoxycytidine induces apoptosis and inhibits tumour growth in vivo of FaDu cells, a specific HPVnegative HNSCC cell line
Source: PLoS One. 2021 Sep 17;16(9):e0253756. doi: 10.1371/journal.pone.0253756 (PMC8448306; doi:10.1371/journal.pone.0253756)

Caspase-3 following 5uM aza 48 hours
Figure 4A

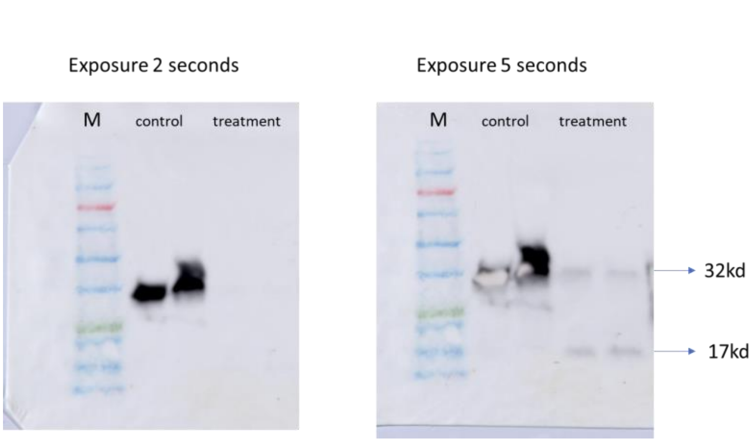

Cas 8 following 5uM aza 48 hours
Figure 4B

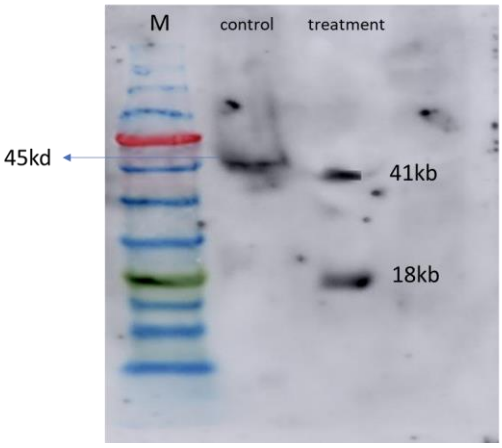

Cas 9 following 5uM aza 48 hours
Figure 4C

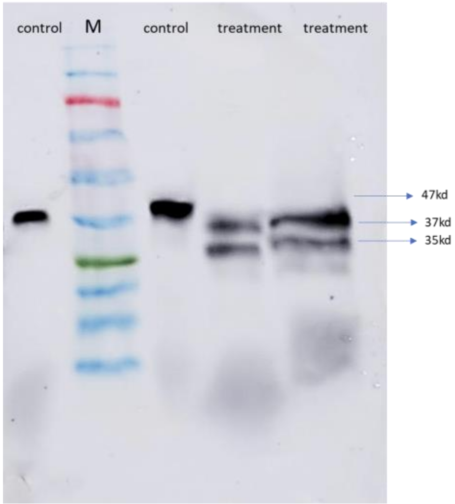

Cleaved PARP following 5uM aza 48 hours
Figure 4D

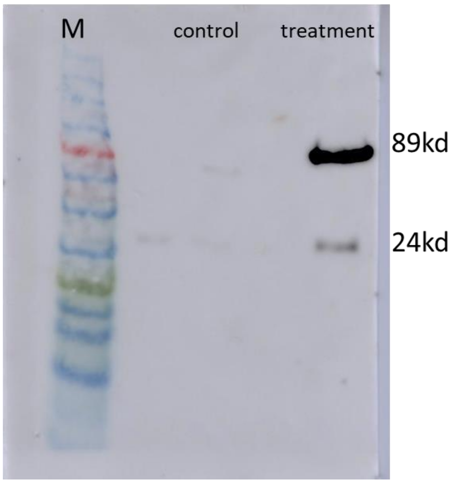

Cytochrome C following 5uM aza 48 hours
Figure 4E

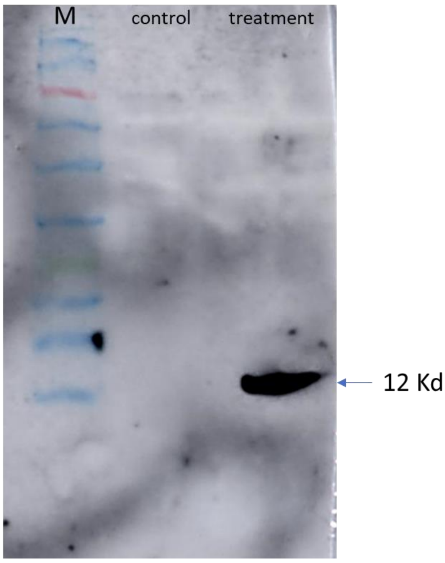

Supplement: S1 Raw images — (PDF) [file pone.0253756.s001.pdf]
